# Peripheral ameloblastoma of the upper gingiva: Report of a case and literature review

**DOI:** 10.4317/jced.51124

**Published:** 2014-04-01

**Authors:** Dario Bertossi, Vittorio Favero, Massimo Albanese, Daniele De-Santis, Manuela Martano, Antongiulio Padovano-di-Leva, Iride De-Florio, Pier F. Nocini, Lorenzo Lo-Muzio

**Affiliations:** 1 Unit of Dentistry and Maxillo-Facial Surgery, University of Verona, Italy; 2 Department of Veterinary Medicine and Animal Productions, University of Naples, Italy; 3 Department of Clinical and Experimental Medicine, University of Foggia, Italy; 4 Department of Medicina clinica, sanita pubblica, scienze della vita e dell`ambiente, University of L`Aquila, L`Aquila, Italy

## Abstract

According to the 2005 histological classification of odontogenic neoplasms by the World Health Organization, ameloblastoma is a benign, locally invasive epithelial odontogenic tumor of putative enamel organ origin. There are four distinct subgroups in which this neoplasm can be gathered: the solid/multicystic type, the unicystic type, the desmoplastic and the peripheral type. Peripheral ameloblastoma is believed to be the rarest subgroup, making up for 2 to 10% of all ameloblastomas. From its first description by Kuru in 1911 to date, less than 200 cases of PA have been described in literature. PAs commonly affect the mandible, in the maxilla the most common location is the soft palatal tissue of the tuberosity area. The present report discusses a rare case of PA aroused in the gingiva of upper jaw in a 64-year-old woman. The treatment of the lesion and its immunohistochemical phenotype are described. A review of the literature is also performed, focusing on the epidemiological and pathological aspects of the lesions and their implications on the therapy.

** Key words:**Peripheral ameloblastoma, upper gingiva, ameloblastoma.

## Introduction

According to the 2005 histological classification of odontogenic neoplasms by the World Health Organization (WHO) (1-3), ameloblastoma is a benign, locally invasive epithelial odontogenic tumor of putative enamel organ origin. Account for about one percent of all oral tumors and about 11% of odontogenic tumors (4). There are four distinct subgroups in which this neoplasm can be gathered: the solid/multicystic type, the unicystic type, the desmoplastic and the peripheral type (1). Peripheral ameloblastoma (PA) is believed to be the rarest subgroup, making up for 2 to 10% of all ameloblastomas (2,5). It was first described by Kuru in 1911 (6). To date, less than 200 cases of PA have been described in literature (7,8). PAs commonly affect the mandible (3,9-12), especially the lingual gingiva in the premolar region (2,13), followed by the anterior region (2,3). In the maxilla, the most common location is the soft palatal tissue of the tuberosity area (2,5). The present report discusses a rare case of PA aroused in the gingiva of upper jaw.

## Case Report

**Figure 1:**
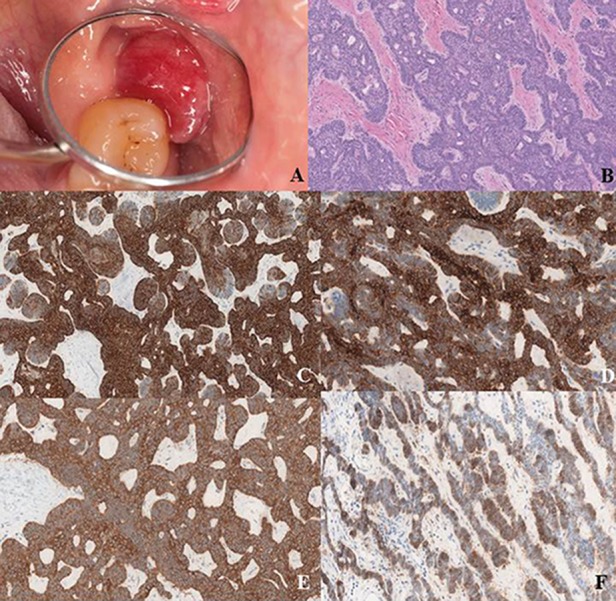
a) Macroscopic aspect of the lesion; b) Hematoxylin-eosin, 40X. The tumor shows a plexiform pattern. It consists of island and strands of odontogenic epithelium within a fibrous stroma. The basal cells of these stands are columnar, hyperchromatic and lined up in a palisaded fashion; c) 40X. Tumor positivity for CK14; d) 40X. Tumor positivity for CK19; e) 40X. Tumor positivity for CKAE1-3; f) 40X. Expression of podoplanin is evident in peripheral cuboidal neoplastic cells of tumor nests and it is expressed slightly in central stellate reticulum-like cells.

A 64-year-old Caucasian woman referred to our Unit with a 2-month history of a tender lesion on the vestibular gingiva of the posterior left maxillary region. No pain or bleeding associated to the lesion was reported. The patient reported previous history of surgically treated breast cancer. She affirmed a smoking history of 5 cigarettes per day in the last 40 years and occasional alcohol consumption. At the clinical examination, there was a sessile mass of about 1 cm distally to the second molar. The mass was dark red and ulcerated (Fig. 1). The consistence was tender; there were no bleeding or pain on palpation. No other lesions in the oral cavity and in the oropharynx, or abnormalities in the head and neck district were reported. CT scan, MRI scan and an incisional biopsy were subsequently performed. Imaging described a 19mm x 15mm x 22 mm mass arising from the posterior alveolar edge of the left maxillary bone, protruding into the vestibule with no signs of bone erosion, or muscular infiltration with regard to the internal and external left pterygoid muscles. No lymphadenopathies were described. The incisional biopsy assessed the presence of a plexiform structured epithelial neoplasm, with basaloid and squamous components; immunohistochemistry showed strong positivity for anticytokeratin antibodies MNF-116, a mild positivity for podoplanin and negativity for calretinin. The overall pattern was suggestive for peripheral ameloblastoma. The patient subsequently underwent the resection of the lesion with surrounding bone tissue and the extraction of the second molar using an intraoral access; a Bichat flap was used for closure. The final pathologic diagnosis was peripheral ameloblastoma, with a plexiform pattern consisting of islands and strands of odontogenic epithelium within a fibrous stroma (Fig. 1). The tumor resulted to be strongly positive for CK14 (Fig. 1) and diffusely positive for CK19 (Fig. 1) and CKAE1-3 (Fig. 1). Ki-67 labeling index was 2%. Moreover, expression of podoplanin was evident in peripheral cuboidal neoplastic cells of tumor nests, whereas it was expressed slightly in central stellate reticulum-like cells (Fig. 1). No complications were observed during recovery. The patient was dismissed three days after surgery. No recurrence of disease was observed after 2-year follow-up.

## Discussion

Epidemiological data extrapolated from the literature assess the maximum incidence of PA in the sixth decade of life, with a male/female ratio amounting to 1,9:1, with an average age of presentation of 52,9 years in males and 50,6 years in females (10). PA seems to be more male-predominant and to occur at a higher age than its intraosseous counterpart (M/F 1,14:1; 35 years) (14,15). The most frequent onset site is the mandibular premolar region, followed by the anterior mandibular region and by the tuber maxillae. Roughly 7 cases of PA out of 10 occur in the lower jaw (2). Review of the English language literature between 1987 to 1999, disclosed only 13 in the maxillary area (12). In their review of 160 PA cases published in English or in Japanese language (74 cases) Philipsen *et al*. reported 46 cases (29.1%) in the maxilla and only 23 cases (8 in not-japanese patients) involving the upper gingiva (2). However other few cases were reported in literatura ( Table 1).

**Table 1:**
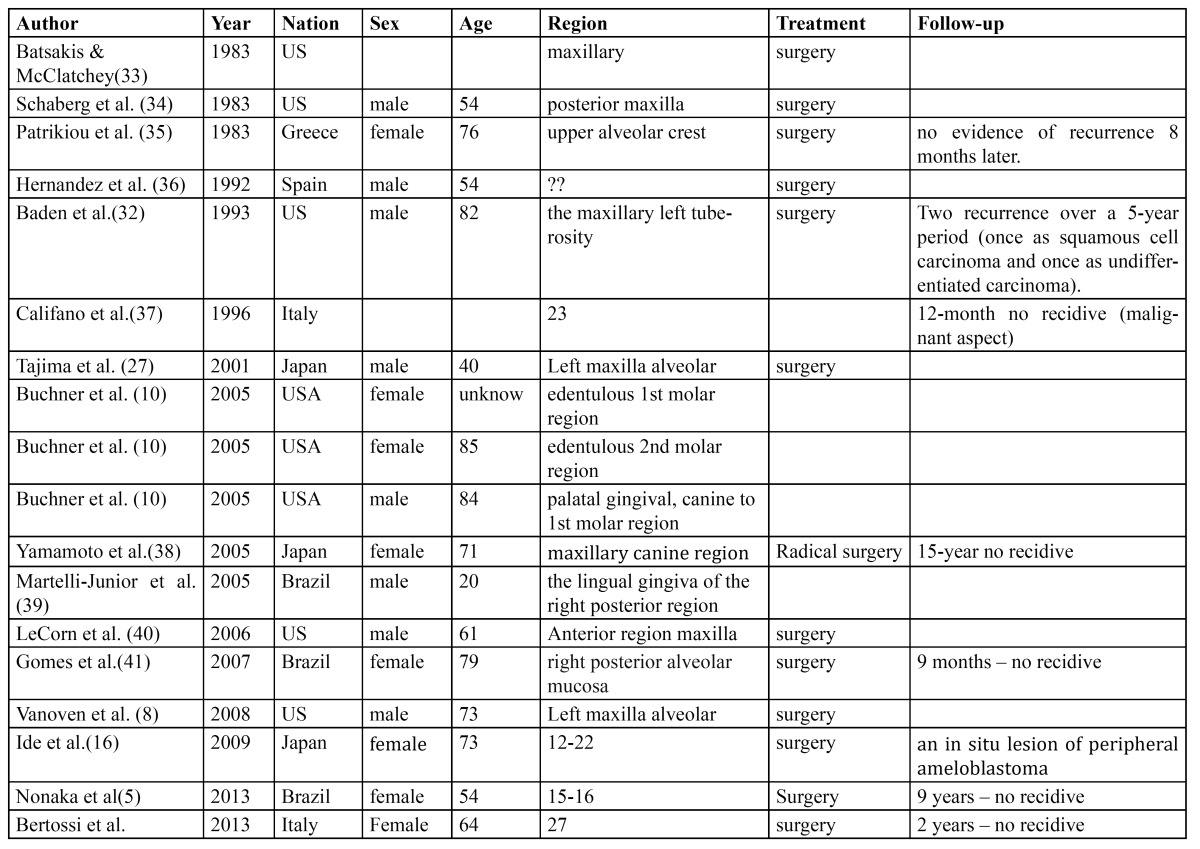
Reported cases of peripheral ameloblastoma of upper gingiva.

The actual histogenesis of PA is still controversial. Two main theories about the cellular origin of PA have been developed. Some tumors, being located completely within the gingival connective tissue with no contact to the surface epithelium or divided from the surface epithelium by a connectival band, are suggestive to derive from the “Serre`s pearls”, that is to say from the extraosseous cellular residuals of the dental lamina (2,3,8). On the other hand, many other cases of PA present themselves in a very close relationship with the surface epithelium. Ide *et al*. described a case of PA in which multiple sectioning failed to detect any epithelial nests in the submucosa, in fact describing an in-situ lesion that seems to provide a remarkable evidence that at least a share of cases of PA take origin from the surface epithelial layer (16,17). Multifocal growth of PA has also been described (18).

The macroscopic presentation of PA is usually that of a solid mass, either sessile or pedunculated, which may be in color from pink to dark red (8). The surface of the mass may be smooth, granular or warty (8). Minute cystic spaces filled with fluid may be found when the mass is cut. Dimensions rarely exceed 2 cm in diameter (8). The microscopic aspect of PA is characterized by ameloblastic growth within a squamous epithelial layer, being the tumor composed by nests of loosely connected cells (19). Another interesting issue about the histology of PA lies in its similarity with basal cell carcinoma (BCC). Some Authors tend to consider the two lesions as the same entity, arguing that they share some peculiar aspects such as the proliferation of basal cells and its island-like arrangement with an important presence of fibrous stroma (20). However, if immunohistochemical analysis is considered, the patterns of positivity for cytokeratins is different between PA and BCC, rather suggesting a greater similarity between PA and its intraosseous counterpart and thus an origin of the lesion from odontogenic epithelial remnants and not from the basal cell layer of the gingival epithelium (3). PA is positive for cytokeratin 19, whereas the opposite is true for BCC (8). A correct distinction among PA and BCC is of pivotal importance, since the treatment of the two lesions greatly differs in the required radicality. The debate on the histogenesis, as assessed previously, is still open and challenging (19,21,22). In literature there are four reported cases of a Peripheral desmoplastic ameloblastoma (23-25).

Apart from BCC, differential diagnosis for PA must consider a range of mucosal and submucosal lesions that may occur in the oral cavity, such as pyogenic granuloma, peripheral ossifying fibroma, peripheral giant cell granuloma, odontogenic gingival epithelial hamartoma. Reactions to ill-fitting dentures and post-inflammatory lesions like fissuratum epulis and inflammatory papillary hyperplasia also have to be considered. PA showing continuity with surface epithelium should as well be differentiated from epithelial neoplasms such as squamous cell carcinoma or verrucous carcinoma (26,27).

As far as imaging is concerned, in most cases there is no evidence of bony infiltration. A cortical bone erosion or a local depression, described as cupping or saucerization, may occasionally be described (2,3,5,10,28). The lack of infiltration may be explained through the existence of a fibrous barrier surrounding the lesion generated by the gingiva and the periosteum. This aspect of the biological behavior of PA makes this pathology greatly different from intraosseous ameloblastoma, which conversely is characterized by a high degree of bony erosion and marrow infiltration, with great implications onto the treatment plan (15).

The treatment of choice for PA is the surgical excision with proper disease-free margins (9,29). No extensive radical treatment is usually required. Although the role of radiation therapy in the treatment of ameloblastomas has been investigated (30), the low occurrence and the peculiar non-aggressive behavior of PA seem to discourage this treatment option. The biological evolutional behavior of PA is characterized by a frequent tendency to recurrence [16%-20%], however inferior to the recurrence rate of intraosseous ameloblastoma (2). It is not clear whether the recurrence rate is an actual feature of the lesion or is rather to be due to incomplete removal of the primary mass. Literature describes a few cases of PA with malignant characteristics (31). These cases presented either with primary and metastatic differentiated benign-appearing lesions or with dedifferentiated lesions. Long-term follow-up is therefore mandatory (32).

In conclusion, considering the descriptions of PAs now available in the literature, these lesions seem to be relatively benign pathologies that however should not be considered harmless at all. Some aspects of PA such as, for instance, its histogenesis and its relationships with similar lesions, still require more studies in order to be fully understood.

## References

[1] Gardner DG, Heikinheimo K, Shear M, Philipsen HP, Coleman H (2005). World health organization classification of tumors Pathology and genetics of head and neck tumors.

[2] Philipsen HP, Reichart PA, Nikai H, Takata T, Kudo Y (2001). Peripheral ameloblastoma: biological profile based on 160 cases from the literature. Oral Oncol.

[3] Kishino M, Murakami S, Yuki M, Iida S, Ogawa Y, Kogo M (2007). A immunohistochemical study of the peripheral ameloblastoma. Oral Dis.

[4] Ghom AG (2010). Textbook of oral medicine: Jaypee Brothers Medical Publishers.

[5] Nonaka CF, de Oliveira PT, de Medeiros AM, de Souza LB, Freitas Rde A (2013). Peripheral ameloblastoma in the maxillary gingiva: a case report. N Y State Dent J.

[6] Kuru H (1911). Ueber das adamantinoma. Zentralbl Allg Pathol.

[7] Yamanishi T, Ando S, Aikawa T, Kishino M, Nakano Y, Sasai K (2007). A case of extragingival peripheral ameloblastoma in the buccal mucosa. J Oral Pathol Med.

[8] Vanoven BJ, Parker NP, Petruzzelli GJ (2008). Peripheral ameloblastoma of the maxilla: a case report and literature review. Am J Otolaryngol.

[9] Pogrel MA, Montes DM (2009). Is there a role for enucleation in the management of ameloblastoma?. Int J Oral Maxillofac Surg.

[10] Buchner A, Merrell PW, Carpenter WM (2006). Relative frequency of peripheral odontogenic tumors: a study of 45 new cases and comparison with studies from the literature. J Oral Pathol Med.

[11] Reichart PA, Philipsen HP, Sonner S (1995). Ameloblastoma: biological profile of 3677 cases. Eur J Cancer B Oral Oncol.

[12] Manor Y, Mardinger O, Katz J, Taicher S, Hirshberg A (2004). Peripheral odontogenic tumours--differential diagnosis in gingival lesions. Int J Oral Maxillofac Surg.

[13] el-Mofty SK, Gerard NO, Farish SE, Rodu B (1991). Peripheral ameloblastoma: a clinical and histologic study of 11 cases. J Oral Maxillofac Surg.

[14] Reichart PA, Jundt G (2008). Benign epithelial odontogenic tumors. Pathologe.

[15] Mendenhall WM, Werning JW, Fernandes R, Malyapa RS, Mendenhall NP (2007). Ameloblastoma. Am J Clin Oncol.

[16] Ide F, Mishima K, Miyazaki Y, Saito I, Kusama K (2009). Peripheral ameloblastoma in-situ: an evidential fact of surface epithelium origin. Oral Surg Oral Med Oral Pathol Oral Radiol Endod.

[17] Ide F (2010). Peripheral ameloblastoma of the buccal mucosa. Oral Surg Oral Med Oral Pathol Oral Radiol Endod.

[18] Gardner DG (1977). Peripheral ameloblastoma: a study of 21 cases, including 5 reported as basal cell carcinoma of the gingiva. Cancer.

[19] Tsuneki M, Maruyama S, Yamazaki M, Cheng J, Saku T (2012). Podoplanin expression profiles characteristic of odontogenic tumor-specific tissue architectures. Pathol Res Pract.

[20] Simpson HE (1974). Basal-cell carcinoma and peripheral ameloblastoma. Oral Surg Oral Med Oral Pathol.

[21] Thosaporn W, Iamaroon A, Pongsiriwet S, Ng KH (2004). A comparative study of epithelial cell proliferation between the odontogenic keratocyst, orthokeratinized odontogenic cyst, dentigerous cyst, and ameloblastoma. Oral Dis.

[22] Gonzalez-Alva P, Tanaka A, Oku Y, Miyazaki Y, Okamoto E, Fujinami M (2010). Enhanced expression of podoplanin in ameloblastomas. J Oral Pathol Med.

[23] Bologna-Molina R, Mosqueda-Taylor A, de Almeida-Oslei P, Toral-Rizo V, Martinez-Mata G (2010). Peripheral desmoplastic ameloblastoma: histopathological and immunohistochemical profile of a case. Med Oral Patol Oral Cir Bucal.

[24] Curran AE, Byerly PD (2008). Peripheral desmoplastic ameloblastoma: report of a rare case. J Oral Maxillofac Surg.

[25] Smullin SE, Faquin W, Susarla SM, Kaban LB (2008). Peripheral desmoplastic ameloblastoma: report of a case and literature review. Oral Surg Oral Med Oral Pathol Oral Radiol Endod.

[26] Lopez-Jornet P, Bermejo-Fenoll A (2005). Peripheral ameloblastoma of the gingiva: the importance of diagnosis. J Clin Periodontol.

[27] Tajima Y, Kuroda-Kawasaki M, Ohno J, Yi J, Kusama K, Tanaka H (2001). Peripheral ameloblastoma with potentially malignant features: report of a case with special regard to its keratin profile. J Oral Pathol Med.

[28] Redman RS, Keegan BP, Spector CJ, Patterson RH (1994). Peripheral ameloblastoma with unusual mitotic activity and conflicting evidence regarding histogenesis. J Oral Maxillofac Surg.

[29] Siar CH, Lau SH, Ng KH (2012). Ameloblastoma of the jaws: a retrospective analysis of 340 cases in a Malaysian population. J Oral Maxillofac Surg.

[30] Koukourakis GV, Miliadou A, Sotiropoulou-Lontou A (2011). Ameloblastoma, a rare benign odontogenic tumour: an interesting tumour review targeting the role of radiation therapy. Clin Transl Oncol.

[31] Wettan HL, Patella PA, Freedman PD (2001). Peripheral ameloblastoma: review of the literature and report of recurrence as severe dysplasia. J Oral Maxillofac Surg.

[32] Baden E, Doyle JL, Petriella V (1993). Malignant transformation of peripheral ameloblastoma. Oral Surg Oral Med Oral Pathol.

[33] Batsakis JG, McClatchey KD (1983). Ameloblastoma of the maxilla and peripheral ameloblastomas. Ann Otol Rhinol Laryngol.

[34] Schaberg SJ, Antimarino RF, Pierce GL, Crawford BE (1983). Peripheral ameloblastoma. Report of a case. Int J Oral Surg.

[35] Patrikiou A, Papanicolaou S, Stylogianni E, Sotiriadou S (1983). Peripheral ameloblastoma. Case report and review of the literature. Int J Oral Surg.

[36] Hernandez G, Sanchez G, Caballero T, Moskow BS (1992). A rare case of a multicentric peripheral ameloblastoma of the gingiva. A light and electron microscopic study. J Clin Periodontol.

[37] Califano L, Maremonti P, Boscaino A, De Rosa G, Giardino C (1996). Peripheral ameloblastoma: report of a case with malignant aspect. Br J Oral Maxillofac Surg.

[38] Yanamoto S, Yamabe S, Kawasaki G, Mizuno A (2005). Peripheral Ameloblastoma in the Maxillary Canine Region. Asian J Oral Maxillofac Surg.

[39] Martelli-Junior H, Souza LN, Santos LA, Melo-Filho MR, De Paula AM (2005). Peripheral ameloblastoma: a case report. Oral Surg Oral Med Oral Pathol Oral Radiol Endod.

[40] LeCorn DW, Bhattacharyya I, Vertucci FJ (2006). Peripheral ameloblastoma: a case report and review of the literature. J Endod.

[41] Gomes CC, Garcia BG, Gomez RS, de Freitas JB, Mesquita RA (2007). A clinical case of peripheral ameloblastoma. Braz J Oral Sci.

